# Time delay and associated mortality from negative smear to positive Xpert MTB/RIF test among TB/HIV patients: a retrospective study

**DOI:** 10.1186/s12879-018-3656-x

**Published:** 2019-01-07

**Authors:** Miguelhete Lisboa, Inês Fronteira, Estefano Colove, Marques Nhamonga, Maria do Rosário O. Martins

**Affiliations:** 1 Centro de Investigação Operacional da Beira (CIOB), Instituto Nacional de Saúde (INS), Rua Correia de Brito #1323 – Ponta-Gea, Beira, Mozambique; 2 Global Health and Tropical Medicine, Instituto de Higiene e Medicina Tropical (IHMT), Universidade NOVA de Lisboa (UNL), Rua da Junqueira N° 100 |, 1349-008 Lisbon, Portugal

**Keywords:** TB/HIV co-infection, Negative smear microscopy, GeneXpert MTB/RIF, Delay TB diagnosis & treatment, TB mortality, Beira-Mozambique

## Abstract

**Background:**

The GeneXpert MTB/RIF Assay (Xpert®) is known to be a feasible, effective and a hopeful tool for rapid tuberculosis (TB) diagnosis and treatment. However, little is known about the time delay caused by initial negative sputum smear microscopy (NSSM), but consecutive positive Xpert TB test (PXTBt) and its association with TB mortality in resource-constrained settings. We aimed to estimate the median time delay between initial NSSM but consecutive PXTBt and TB treatment initiation and its association with TB mortality among TB/HIV co-infected patients in Beira, Mozambique.

**Methods:**

we used data from a retrospective cohort study of TB/HIV co-infected patients in six TB services in Beira city. The study included all patients that tested NSSM, followed by a PXTBt in the six health centers with TB services during the year 2015. Data were extracted from the laboratory and TB treatment registers. To assess the difference in median time delays between groups, Mann-Whitney and Kruskal-Wallis tests were computed. To analyze the associations between the time delays and TB mortality, logistic regression model was used.

**Results:**

Among the 283 patients included in the study, median (IQR) age was 31 (17) years, 59.0% were males, 57.6% in the WHO clinical fourth stage of HIV. The median (IQR) values for diagnostic delay, treatment delay and total time delay was 10 (9) days, 13 (12) days and 28 (20) days, respectively. For TB/HIV co-infected patients who tested negative for smear microscopy initially, a total time delay of one month or longer was associated with high mortality (aOR = 12.40, 95% CI: 5.70–22.10).

**Conclusion:**

Our study indicates that delays in TB diagnosis and treatment resulting from initial NSSM, but consecutive PXTBt are common in Beira city and are one of the main factors associated with TB mortality among TB/HIV co-infected patients. Applying GeneXpert assay as gold standard for HIV-positive patients with suspected pulmonary TB or replacing the sputum smear microscopy by Xpert assay and its availability within 24 h is urgently needed to ensure early diagnosis and treatment, and to maximize the impact of the few resources available in the country.

## Background

Tuberculosis (TB) diagnosis and treatment delays increase the risk of spreading TB in the community and in health facilities [1–3], developing severe disease and death, particularly in TB/HIV co-infected patients [1, 3, 4]. Additionally, TB remains one of the most common opportunistic infections and the major cause of morbidity and mortality in people living with HIV [5–7]. Of the 10.4 million new TB cases worldwide in 2016, an estimated 10% where associated with the Human Immunodeficiency Virus (HIV) and 22.3% of all TB deaths were HIV positive [8].

Mozambique ranks in 14th place among the 30 countries with the highest burden of TB, with incidence rates estimated at 551/100,000 inhabitants in the general population and 284/100,000 inhabitants in the HIV-infected population. The TB/HIV co-infection rate in Mozambique is estimated at 44%, being one of the highest in the world [8].

In 2015, Beira city reported a TB incidence rate estimated at 659/100,000 inhabitants. Among the 3034 new TB cases in 2015, about 63% tested positive for HIV demonstrating that both, TB incidence and TB/HIV co-infection rates in Beira are above the national average [9].

TB/HIV co-infection represents a huge challenge for TB control, particularly in low-income countries, such as Mozambique. One of the main strategies for TB control is to reduce transmission through early detection and rapid administration of proper anti-TB treatment [10, 11]. However, several studies and systematic reviews of literature have shown that there are both patient delays and health care system delays that hinder TB diagnosis and treatment [12–16].

**Fig. 1:**
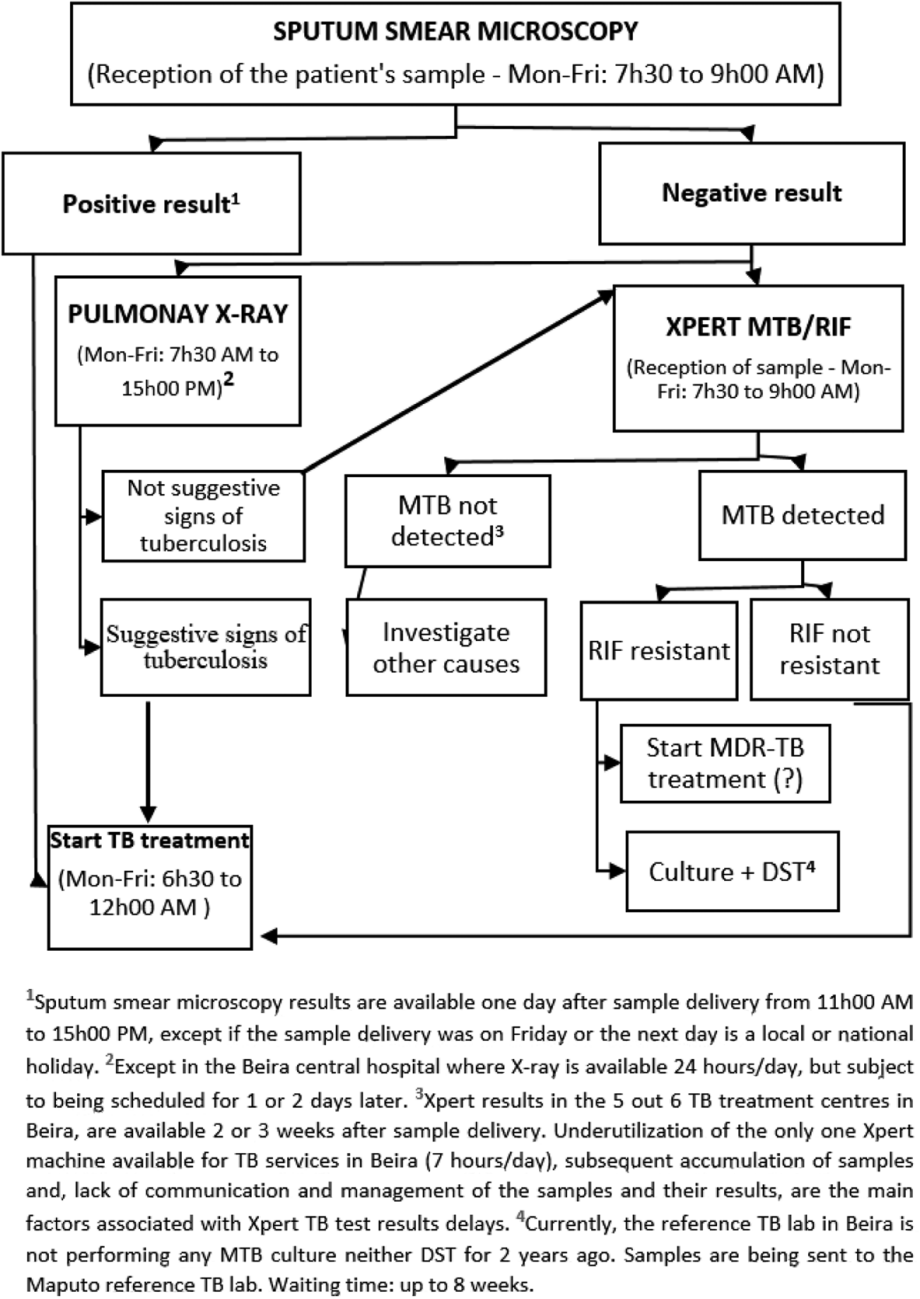
Current flowchart for TB diagnosis in Beira city

While the World Health Organization’s recommendation [17] from 2014 has incorporated GeneXpert as gold standard for the diagnosis of HIV positive adults with suspected TB, Mozambique’s guidelines are still lagging behind. In line with the national guideline for the implementation of GeneXpert MTB/RIF (Xpert) [18] health facilities without GeneXpert should perform acid-fast bacilli smear microscopy as the first method of choice to diagnose TB. Patients with HIV or other immunosuppressive conditions (diabetes, pregnancy, etc.) that are suspected to have TB, are only referred to GeneXpert assay, after receiving negative results for the acid-fast bacilli smear microscopy [18] Fig. 1.

On the other hand, using the signs and symptoms [19], pulmonary X-ray [20, 21] and sputum smear microscopy [22, 23] for the diagnosis of pulmonary TB in HIV-infected patients has been appointed to be inefficient and considered as important risk factors for delays in TB diagnosis and treatment, particularly among TB/HIV co-infected patients [24, 25].

However, little is known about the possible time delays in TB treatment initiation that result from initial negative sputum smear microscopy in TB/HIV co-infected persons. As further research is needed to show the extent to which much morbidity and mortality is associated with the time delays in TB diagnosis and treatment initiation among TB/HIV co-infected patients in Beira, our study aimed to estimate the median time delay in TB diagnosis and treatment, as well as TB mortality associated with both types of delays among patients co-infected with TB/HIV.

## Methods

### Study design, population and sample

This was retrospective cohort study conducted in the all six health centers with TB services in Beira city, from January to December 2015. TB laboratory and treatment registers were retrospectively reviewed. We purposively retrieved only data of all pulmonary TB/HIV co-infected patients who had an initial negative sputum smear microscopy; but were subsequently found to have positive GeneXpert MTB test. Therefore, patients with previous positive GeneXpert MTB test, positive sputum smear microscopy and extra pulmonary TB, were excluded from the study.

In Mozambique, HIV patients start to take HAART when they are at WHO stage 3 or 4 regardless CD4+ count or at WHO stage 1 or 2 with CD4+ count below 500 cel/ mm^3^. However, currently, Mozambique is piloting a new “test and treat” strategy, by which every HIV positive person is offered HAART. Since 2016, combined fixed dose drugs are available and used accordingly. The recommended first and second choices for adults HIV treatment are TDF + 3TC + EFV or AZT + 3TC + NVP or ABC + 3TC + EFV and AZT + 3TC + LPV/r or TDF + 3TC + LPV/r or ABC + 3TC + LPV/r, respectively [26].

On the other hand, isoniazid, rifampicin, ethambutol, and pyrazinamide are the combined fixed dose drugs used for the treatment of TB. In addition, injectable drugs such as streptomycin, kanamycin, amikacin, ofloxacin, levofloxacin, ethionamide and cycloserine are available drugs for the 2nd line regimen [27].

### Study operational definitions

Time delays under investigation of this study are:

- the delay to diagnosis with GeneXpert assay - considered as difference in days between negative smear microscopy and positive GeneXpert assay
- the delay to treatment initiation – considered as difference in days between positive GeneXpert assay and proper TB drug administration
- the total time delay - considered as difference in days between having a negative smear microscopy and treatment initiation.

### Study site and TB services

The study was carried out in Beira city (the capital of Sofala province) central region of Mozambique. Beira is the 2nd largest city in Mozambique (after Maputo city, capital of Mozambique) located about 1200 km northern of Maputo, with population estimated about 463,442 inhabitants in 2017, which 50.2% are male [28].

Beira city has a referral TB laboratory for the central region of the country, installed in the Beira central hospital (HCB). This laboratory has the ability to culture *M. tuberculosis*, drug sensitivity test, in addition to the diagnosis of TB through Gene Xpert MTB/RIF and smear microscopy based on fluorescence. Additionally, in the primary health care network, there are 6 of the 13 primary healthcare facilities that have TB diagnosis and treatment services, namely: Macurungo, Munhava, Mascarenhas, Nhaconjo, Chingussura and Ponta-Gêa health centers.

**Fig. 2:**
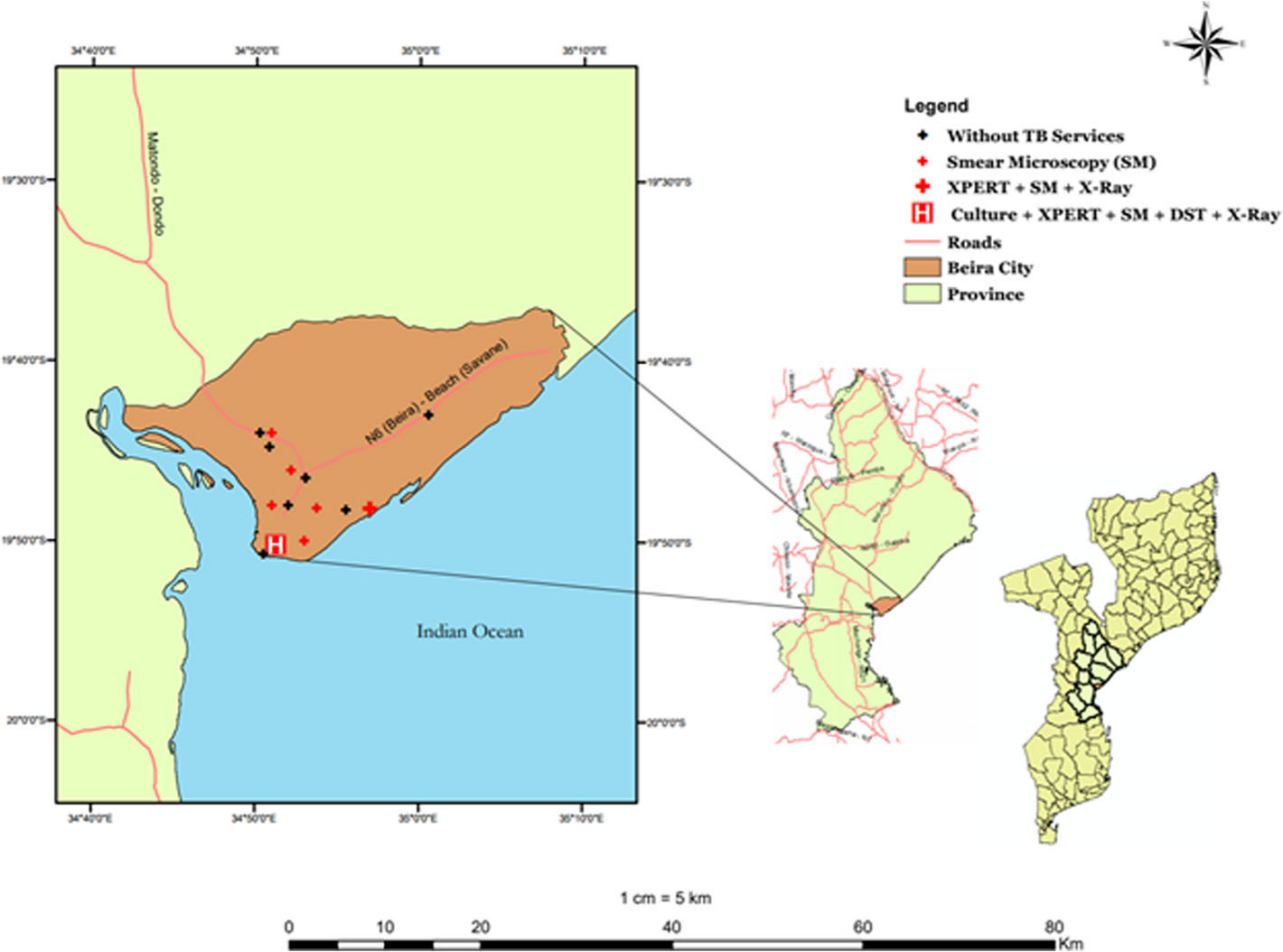
TB services and diagnostic capabilities in Beira city, Mozambique (**designed by the authors**)

Among the primary healthcare facilities above-listed, Ponta-Gêa is the only one out of the six primary healthcare facilities with TB services equipped with GeneXpert MTB/RIF technology, X-ray machine, higher number of high qualified lab technicians, nurses, and medical doctors. Additionally, Ponta-Gêa is the largest TB and HIV center in Beira city. The available Xpert machine in Ponta-Gêa health facility is a 4-module configuration, therefore Xpert sample testing response is also delayed due to sample overload and limited working hours of the TB laboratories Fig. 2.

### Variables and, inclusion or exclusion criteria

Demographic and clinical data such as sex, age, HIV and treatment status, WHO clinical stages of HIV, sputum smear microscopy result, Gene Xpert sputum results, as well as (a) the date recorded for negative smear microscopy, (b) the date recorded for positive GeneXpert assay, (c) the date recorded for anti-Tuberculosis treatment initiation and (d) the treatment outcome, were retrospectively extracted from the laboratory and treatment registration books.

Patients without data in relation to HIV and TB treatment outcome, and/or transferred out to other health facilities out of the Beira city, were excluded from the analysis.

### Data analysis

The collected data were introduced, cleaned and analyzed using Statistical Package for Social Sciences (SPSS) version 20 for Windows. Descriptive statistics: frequencies, percentages, medians and interquartile range were used to present the data. The time delay from negative sputum smear microscopy initially documented, followed by a positive GeneXpert assay that demonstrated pulmonary Tuberculosis to TB treatment initiation was assessed.

To assess the difference in different median time delays between groups we used Mann-Whitney and Kruskal-Wallis nonparametric test. To analyze the associations between the several types of time delays and TB mortality among TB/HIV co-infected patients, logistic regression model was used. We computed adjusted odds ratio (aOR) and its respective 95% confidence interval. We consider a significance level of 5% for all statistical analysis.

## Results

### Patient characteristics

**Fig. 3:**
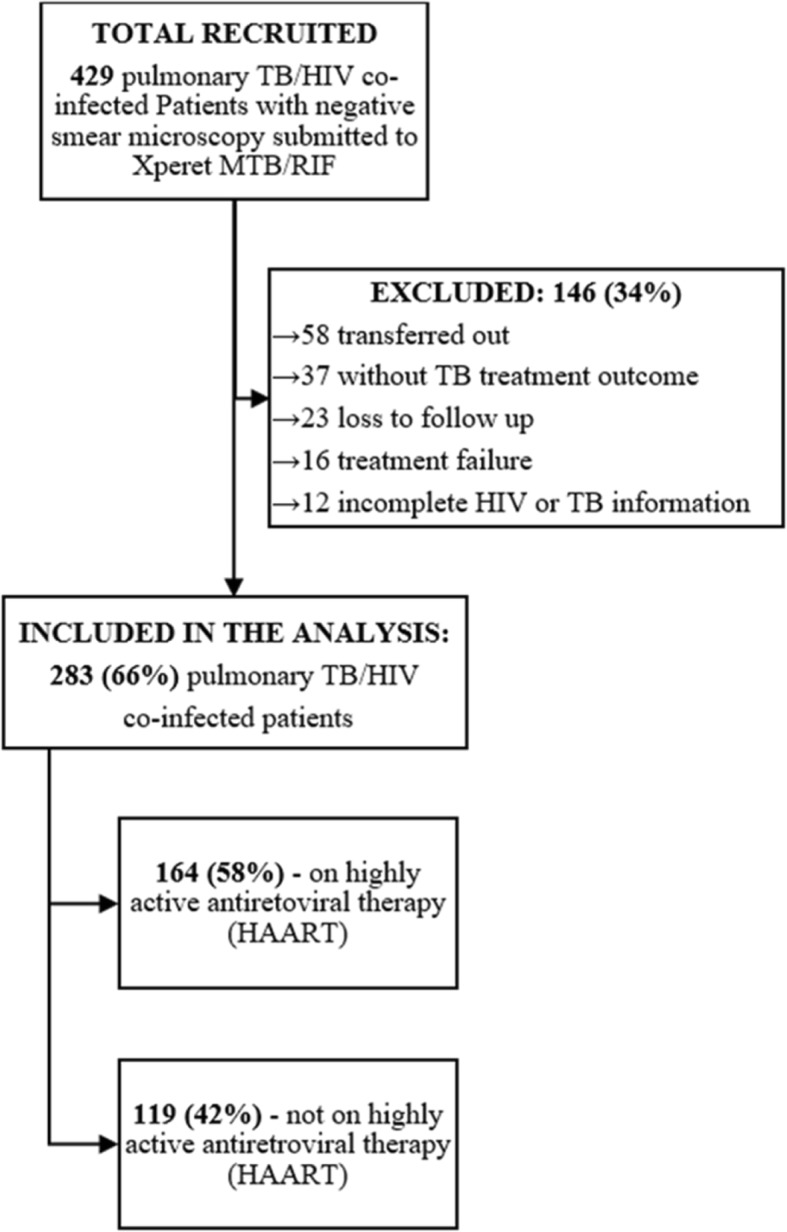
Schematic representation of study population and selection procedure

A total of 428 pulmonary TB/HIV co-infected patients were submitted for Xpert sputum test after negative sputum smear microscopy in Beira city, between January and December 2015. Of these, 283 (66.1%) had complete information and were included in the data analysis. Missing information was related to incomplete treatment outcomes records of 37 patients, incomplete TB diagnosis, age and highly active antiretroviral therapy (HAART) information of 12 patients, lost to follow-up of 23 patients, treatment failure of 16 patients and, 58 transferred out, and hence with unknown TB treatment outcomes Fig. 3.

From the total TB/HIV co-infected patients, 167 (59.0%) were males and 164 (58.0%) were on HAART. The median (interquartile range - IQR) age was 31 [17] years and the most frequent age category was 25–34 years old (39.2%). Forty-nine (17.3%) of the patients had MTB detected and RIF resistant while the remaining were not resistant. The majority, 163 (57.6%) of the TB/HIV co-infected patients had more advanced HIV disease (WHO clinical 4th stage of HIV), 158 (55.8%) died during TB treatment and the remaining completed the TB treatment successfully (Table 1).

**Table 1:** General characteristics of pulmonary TB/HIV co-infected patients with positive Xpert spuum test and registered for treatment in 2015 in Beira city, Mozambique

Characteristics	Number	Percentage (%)
Sex		
Male	167	59.0
Female	116	41.0
Age groups		
15–24 years old	65	23.0
25–34 years old	111	39.2
35–44 years old	50	17.7
45–54 years old	28	9.9
> 55 years old	29	10.2
WHO clinical stages of HIV		
Stage 3	120	42.4
Stage 4	163	57.6
*M.tuberculosis*/rifampicina (MTB/RIF)		
Resistant	49	17.3
Not resistant	234	82.7
Highly active antirretroviral therapy (HAART)		
On HAART	164	58.0
Not on HAART	119	42.0
Treatment outcome		
Died	158	55.8
Completed	125	44.2

*HAART* = Highly active antiretroviral therapy, *WHO* = World health organization

### Median time delays in TB diagnosis and treatment

The total median (IQR) time delay was 28 [20] days. The median (IQR) time delay from the initial negative sputum smear microscopy (NSSM) to the positive Xpert TB test (PXTBt) and, from this point to the TB treatment initiation was 10 [9] days and, 13 [12] days, respectively.

The median time delay from the initial negative sputum smear microscopy (NSSM) to TB treatment initiation is not statistically different between the sex and among the age groups (*p* > 0.05). However, statistically significant differences in the median time delays were observed among the TB treatment centers, between those with successful treatment outcome and those who died and, between patients with WHO clinical 3rd and 4th stages of HIV (*p* < 0.05).

Details about median time delays from NSSM to PXTBt and from PXTBt to TB treatment initiation are given (Table 2).

**Table 2:** Median time delays from negative smear microscopy to TB treatment through positive Xpert test among pulmonary TB/HIV co-infected patients during 2015 in Beira city, Mozambique

Characteristics	Delays in TB diagnosis and Treatment (DAYS)		
	From NSSM to PXTBt	From PXTBt to TB Treatment initiation	From NSSM to TB Treatment initiation
	Median (IQR)	Median (IQR)	Median (IQR)
Sex			
Male	9 (13)	14 (14)	30 (23)
Female	7.5 (8)	13 (11)	26 (19)
*p-value* (Mann-Whitney U)	0.127	0.579	0.364
Age groups			
15–24 years old	7 (7)	13 (13)	27 (21)
25–34 years old	9 (11)	14 (11)	29 (17)
35–44 years old	9 (9)	13 (15)	27 (20)
45–54 years old	12.5 (14)	14 (10)	33.5 (26)
> 55 years old	11 (7)	12 (6)	25 (15)
*p-value* (Kruskal-Wallis)	0.303	0.905	0.585
TB treatment center			
Ponta-Gea	5 (12)	8 (4)	13 (23)
Macurungo	13 (8)	13 (12)	26 (10)
Manga Mascarenha	11 (8)	17 (8)	33.5 (20)
Munhava	11 (10)	15 (12)	32 (12)
Nhaconjo	12 (13)	14 (9)	27 (11)
Chingussura	13 (22)	15.5 (9)	36 (24)
*p-value* (Kruskal-wallis)	0.001	0.001	0.001
TB treatment outcome			
Completed	7 (9)	10 (11)	23 (19)
Died	11 (10)	14.5 (12)	31.5 (20)
*p-value* (Mann-Whitney U)	0.013	0.001	0.001
WHO clinical satage of HIV			
Stage 3	9 (8)	11 (11)	22 (15)
Stage 4	7 (13)	19 (13)	25 (21)
*p-value* (Mann-Whitney U)	0.947	0.002	0.004
Total	10 (9)	13 (12)	28 (20)

*IQR* = Interquartile range, *NSSM* = Negative Sputum Smear Microscopy, *PXTBt* = Positive Xpert TB test

### Tuberculosis mortality and its correlates

The majority of patients (55.8%) died during TB treatment. Estimation results indicated that sex and age groups are not statistically associated with mortality (*p* > 0.05), while time delay in TB diagnosis and treatment increases the probability of TB death.

A patient with TB/HIV co-infection who has a time delay of up to 28 days (aOR = 4.02, 95% CI: 2.31–9.92), when compared to the reference class (≤ 7 days of delay), is 4 times more likely to die from tuberculosis, adjusting for other factors; while a patient with a time delay of more than a month (aOR = 12.40, 95% CI: 5.70–22.10), is over 12 times more likely to die, adjusting for other factors (Table 3).

**Table 3:** Risk factors associated with TB mortality among pulmonary TB/HIV co-infected patients in Beira city, Mozambique

Characteristics	Adjusted Odds Ratio (aOR)	95% Confidence Interval (CI)		*P*-value
		Minimum	Maximum	
Time delays in days [Reference, ≤ 7 days]				
8–14 days	1.11	0.72	3.91	0.097
15–21 days	1.69	1.12	5.59	0.032
22–28 days	4.02	2.31	9.92	0.001
≥ 29 + days	12.40	5.70	22.10	0.001
Sex [Reference, female]				
Male	1.21	0.75	1.95	0.431
Age category [Reference, 15–24 years old]				
25–34 years old	1.09	0.44	2.68	0.850
35–44 years old	1.67	0.69	3.71	0.267
45–54 years old	1.18	0.46	3.02	0.723
> 55 years old	1.23	0.43	3.54	0.705
WHO clinical stages of HIV [Reference, stage 3]				
Stage 4	5.45	3.11	9.56	0.001
HAART [Reference, on HAART]				
Not on HAART	6.86	3.94	11.95	0.001
TB treatment center [Reference, Ponta-Gea]				
Macurungo	1.96	1.29	4.73	0.009
Manga Mascarenha	2.08	1.51	5.18	0.001
Munhava	3.13	1.93	6.37	0.001
Nhaconjo	1.89	1.31	4.92	0.003
Chingussura	2.71	1.74	5.03	0.006

*aOR* = adjusted Odds Ratio, *HAART* = Highly active antiretroviral therapy

The WHO clinical stages of HIV infection, highly active antiretroviral therapy (HAART) status and localization of TB treatment centers are also associated with mortality. Thus, a patient in the WHO clinical 4th stage of HIV (aOR = 5.45, 95% CI: 3.11–9.56) when compared with 3rd stage of HIV is 5 times more likely to die from TB adjusting for other factors. Similarly, patient that is not on HAART (aOR = 6.86, 95% CI: 3.94–11.95), when compared with those on HAART, is 6 times more likely to die from TB adjusting for other factors.

Additionally, patients treated in Ponta-Gea TB center (the reference group) are less likely to die from TB, adjusting for other factors.

## Discussion

One of the main findings of this retrospective study is the median (IQR) time delay of 28 [20] days in the TB diagnosis and treatment estimated from the initial negative sputum smear microscopy (NSSM) to TB treatment initiation. Similar studies have found a median (IQR) health system delay of 62 (83) days across all health facilities in Beira city among all TB suspected patients [16].

Despite the political commitment and increased governmental efforts in the last 10 years, the availability of TB services still strives to meet demands: based on the national labor law [29], the TB labs and TB treatment centers within the public health facilities are open from 7:30 AM to 3:30 PM and 7:30 AM to 12:00 PM, respectively, during working days. TB treatment centres and sputum sample examination are not performed on weekends or holidays.

Additionally, in line with the national guideline for the implementation of GeneXpert MTB/RIF [18], patients with HIV suspected to have TB within health facilities without GeneXpert MTB/RIF machine are only referred to Xpert assay after receiving negative results for the acid-fast bacilli smear microscopy. Unfortunately, as Xpert machine is only available in one out of the six primary healthcare facilities with TB services and is a 4-module configuration, the Xpert sample testing response is also delayed due to sample overload and limited working hours of the TB laboratories.

However, despite the ongoing contribution for TB control in resource-limited settings, using the signs and symptoms [19], pulmonary X-ray [20, 21] and sputum smear microscopy [22, 23] for the diagnosis of pulmonary TB in HIV-infected patients has been reported to be inefficient and an important risk factors for delays in TB diagnosis and treatment [24, 25].

Therefore, as the TB/HIV co-infection rate in Beira city is estimated at 63% [9] and most of patients present their selves to the health facility at advanced stage of HIV infection, which has been associated with a negative sputum smear microscopy [30, 31], the use of the Xpert MTB/RIF within the primary healthcare facilities (currently: one out six) at first attempt to TB diagnosis among TB/HIV co-infected patients in Beira city should be promoted and available 24 h per day to ensure early pulmonary TB diagnosis and prompt initiation of proper treatment.

Our data also demonstrate that the mortality rate was 55,8% among TB/HIV co-infected patients who had negative sputum smear microscopy (NSSM) at first attempt to rule out TB infection followed by positive Xpert TB test. The median time delay of 4 weeks or more in TB diagnosis and treatment, the WHO clinical 4th stage of HIV and patient not on HAART were associated with mortality. Additionally, primary healthcare facilities like Munhava, Chingussura and Manga Mascarenha, where the patients’ flow is greater, including TB/HIV patients and, are distant from Ponta-Gea health facility where Xpert TB test are performed, were also associated with higher mortality.

Similar results have been reported that delays in TB diagnosis & treatment and HAART initiation, advanced HIV infection and high risk associated with tuberculosis-immune reconstitution inflammatory syndrome are associated with high mortality among TB/HIV co-infected patients [30, 32–37].

Therefore, as Xpert assay is known to be faster and better sensitivity and specificity than sputum smear microscopy and, can effectively be used in resource-limited settings [23, 38–41] its use as a first tool of TB diagnosis should be promoted within the primary healthcare facilities in Beira to simplify patients’ access to early and accurate diagnosis, thereby potentially decreasing severe TB cases and, associated TB mortality resulted from delays in TB diagnostic and treatment.

The main weakness of the study design is the use of retrospective data merely from TB/HIV co-infected patients with advanced HIV infection (WHO clinical 3rd and 4th stages of HIV) who are more likely to have negative sputum smear microscopy and high mortality. Additionally, the study design was not adequate to determine the time and causes of mortality among TB/HIV co-infected patients, which should provide further insight into some findings. However, a multicentre prospective cohort study [30] and a systematic review and meta-analysis [35] have documented that most deaths occurred within the first 3 months of HAART initiation before anti-TB drugs and within the first 2 weeks of anti-TB therapy.

## Conclusions

Our study suggested that time delays in TB diagnosis and treatment resulting from initial negative sputum smear microscopy, but consecutive positive GeneXpert MTB/RIF test are common in Beira city and, is one of the main factors associated with mortality among TB/HIV co-infected patients. Applying GeneXpert assay as gold standard for HIV-positive patients with suspected pulmonary TB or replacing the sputum smear microscopy by Xpert assay at all, and its availability within 24 h is urgently needed to ensure early diagnosis and treatment, and to maximize the impact of the few resources available in the country. This approach can also help in reducing the transmission of tuberculosis and the development of severe cases of TB, as well as in reducing TB mortality related to the delay in diagnosis and treatment.

## Abbreviations

aOR: Adjusted odds ratio
CIOB: Centro de investigação operacional da beira
FCG: Calouste gulbenkian foundation
EC: Estefano colove
HAART: Highly active antirretroviral therapy
HIV: Human immunodeficiency virus
IF: Inês fronteira
INS: Instituto Nacional de Saúde
IHMT: Instituto de Higiene e Medicina Tropical
IQR: Interquartile range
MROM: Maria do Rosário Oliveira Martins
MN: Marques Nhamonga
ML: Miguelhete Lisboa
NSSM: Negative Sputum Smear Microscopy
PXTBt: Positive Xpert TB test
TB: Tuberculosis
UNL: Universidade Nova de Lisboa
WHO: World Health Organization
